# Complete Resolution of Atypical Paraneoplastic Pemphigus Following Treatment With Dupilumab

**DOI:** 10.7759/cureus.81033

**Published:** 2025-03-23

**Authors:** Gabriela Pinero-Crespo, Nirav Shah, Skylar Klager, Catherine Kowalewski

**Affiliations:** 1 Dermatology and Cutaneous Surgery, University of South Florida Morsani College of Medicine, Tampa, USA; 2 Dermatology, James A. Haley Veterans' Hospital, Tampa, USA

**Keywords:** atypical presentation, direct immunofluorescence, dupilumab, paraneoplastic pemphigus, refractory pruritus

## Abstract

Paraneoplastic pemphigus (PNP) is a rare autoimmune mucocutaneous blistering condition associated with underlying malignancies. While severe stomatitis is usually a hallmark feature of PNP, it may present with the absence of oral manifestations. Proposed diagnostic criteria include the presence of mucosal lesions with or without cutaneous involvement, concomitant internal neoplasm, anti-plakin autoantibodies, histopathology with acantholysis and/or lichenoid interface changes, and direct immunofluorescence (DIF) with intercellular and/or basement membrane deposition of immunoglobulin G (IgG) or complement component-3 (C3). Management of PNP traditionally involves immunosuppressants; however, prognosis remains poor.

This report presents a 77-year-old male patient with an ongoing, intensely painful, and pruritic rash with diffuse scaling, erythema, crust, bullae, and erosions covering 90% body surface area that notably lacked mucosal involvement. Biopsies revealed acantholysis with mixed infiltrate lichenoid interface dermatitis and DIF with 3+ intercellular and basement membrane deposition of C3 and IgG. Additional workup revealed elevated desmoglein-1 and bullous pemphigoid antigen-1 (BPAG1/BP230) autoantibodies and intramucosal gastrointestinal adenocarcinoma, satisfying the diagnostic criteria for PNP.

Due to his history of type 2 diabetes mellitus, chronic non-healing ulcers, and untreated malignancy, he was a poor candidate for first-line immunosuppressants. Additionally, the patient experienced significant pruritus resistant to topical corticosteroids and oral antihistamines. For these reasons, dupilumab was elected to control the patient's pruritus. After two months, there was significant improvement in skin lesions, and after four months, there were complete resolution of pruritus and 100% clearance of skin lesions.

This report highlights a unique and previously unreported case of complete resolution of atypical PNP following empiric treatment with dupilumab, a monoclonal antibody against interleukin 4/13 receptor-α.

## Introduction

Paraneoplastic pemphigus (PNP) is a rare autoimmune mucocutaneous blistering condition associated with internal malignancies, including carcinomas, sarcomas, and hematologic malignancies [1]. The classic clinical feature is severe stomatitis [2]; however, there are reports of atypical forms of PNP without mucosal lesions [3].

Revised diagnostic criteria include the presence of mucosal lesions with or without cutaneous involvement, concomitant internal neoplasm, anti-plakin autoantibodies, histopathology showing acantholysis and/or lichenoid interface changes, and direct immunofluorescence (DIF) with intercellular and/or basement membrane deposition of immunoglobulin G (IgG) and/or complement component-3 (C3) [4].

Management of PNP involves treating the underlying neoplasm, mucocutaneous symptoms, and wounds. Treatment traditionally involves immunosuppressants; however, prognosis remains poor, with mortality ranging from 75% to 90% [5]. The main causes of death include bronchiolitis obliterans, infection, and underlying malignancy [5].

This report highlights a unique and previously unreported case of complete cutaneous resolution of atypical PNP, without mucosal involvement, following empiric treatment with dupilumab, a monoclonal antibody against interleukin 4/13 receptor-α, initially used to manage pruritus. 

This case was previously presented as a poster presentation at the Florida Academy of Dermatology 2024 Annual Meeting on June 28-30, 2024.

## Case presentation

A 77-year-old male patient with type 2 diabetes mellitus (T2DM)-associated ulcers and right above-the-knee amputation initially presented for an ongoing painful, diffuse, blistering eruption without mucosal involvement. He reported no new medications. His only known allergies included a rash with neomycin.

**Figure 1:**
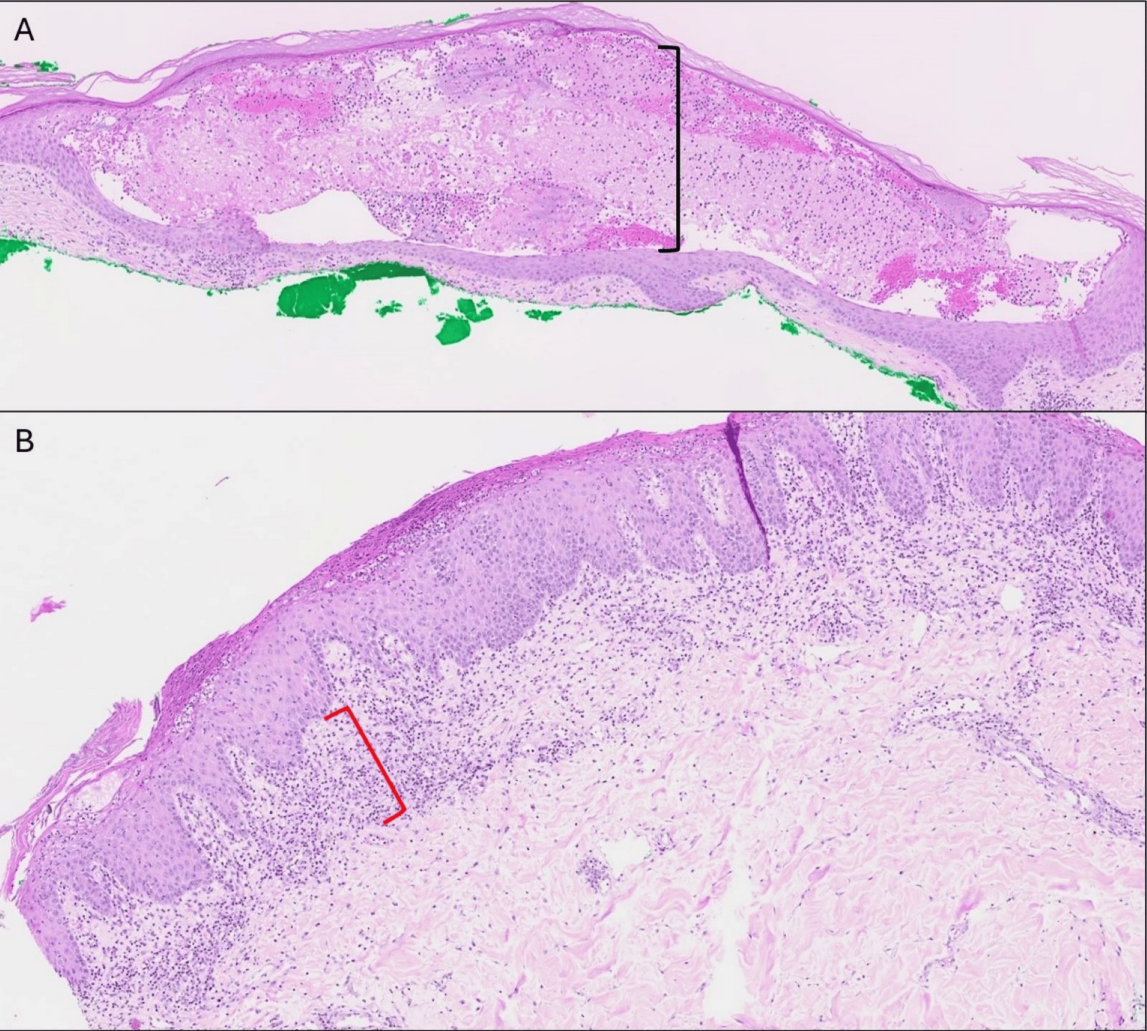
(A) Skin biopsy of paraneoplastic pemphigus at 1× magnification showing acantholysis. (B) Skin biopsy of paraneoplastic pemphigus at 2× magnification showing lichenoid interface dermatitis. Black bracket indicates acantholysis or separation of keratinocytes within the epidermis. Red bracket indicates lichenoid interface dermatitis or a band of inflammatory cells in the upper dermis near the dermal-epidermal junction.

Initial biopsies revealed acantholysis with mixed infiltrate lichenoid interface dermatitis (Figure 1A, 1B), one biopsy with negative DIF, and another with DIF positive for intercellular C3. Diagnosis favored acute generalized exanthematous pustulosis (AGEP) or pemphigus foliaceus (PF). Serology revealed elevated desmoglein-1 and bullous pemphigoid antigen-1 (BPAG1/BP230) autoantibodies with normal desmoglein-3 and bullous pemphigoid antigen-2 (BPAG2/BP180). Suspicion for PNP prompted an underlying malignancy workup. A positron emission tomography (PET) scan showed a hypermetabolic rectal soft tissue lesion concerning for malignancy. Subsequent colonoscopy and biopsy confirmed intramucosal rectal adenocarcinoma in the background of a tubulovillous adenoma. After resection, margins remained positive, but the patient declined further surgical or medical treatment.

**Figure 2:**
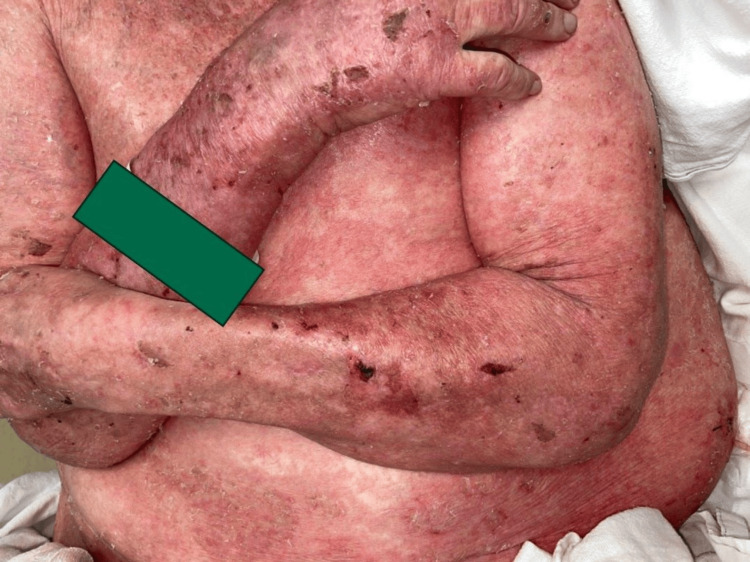
Paraneoplastic pemphigus involving the arms, chest, and abdomen, pre-treatment.

**Figure 3:**
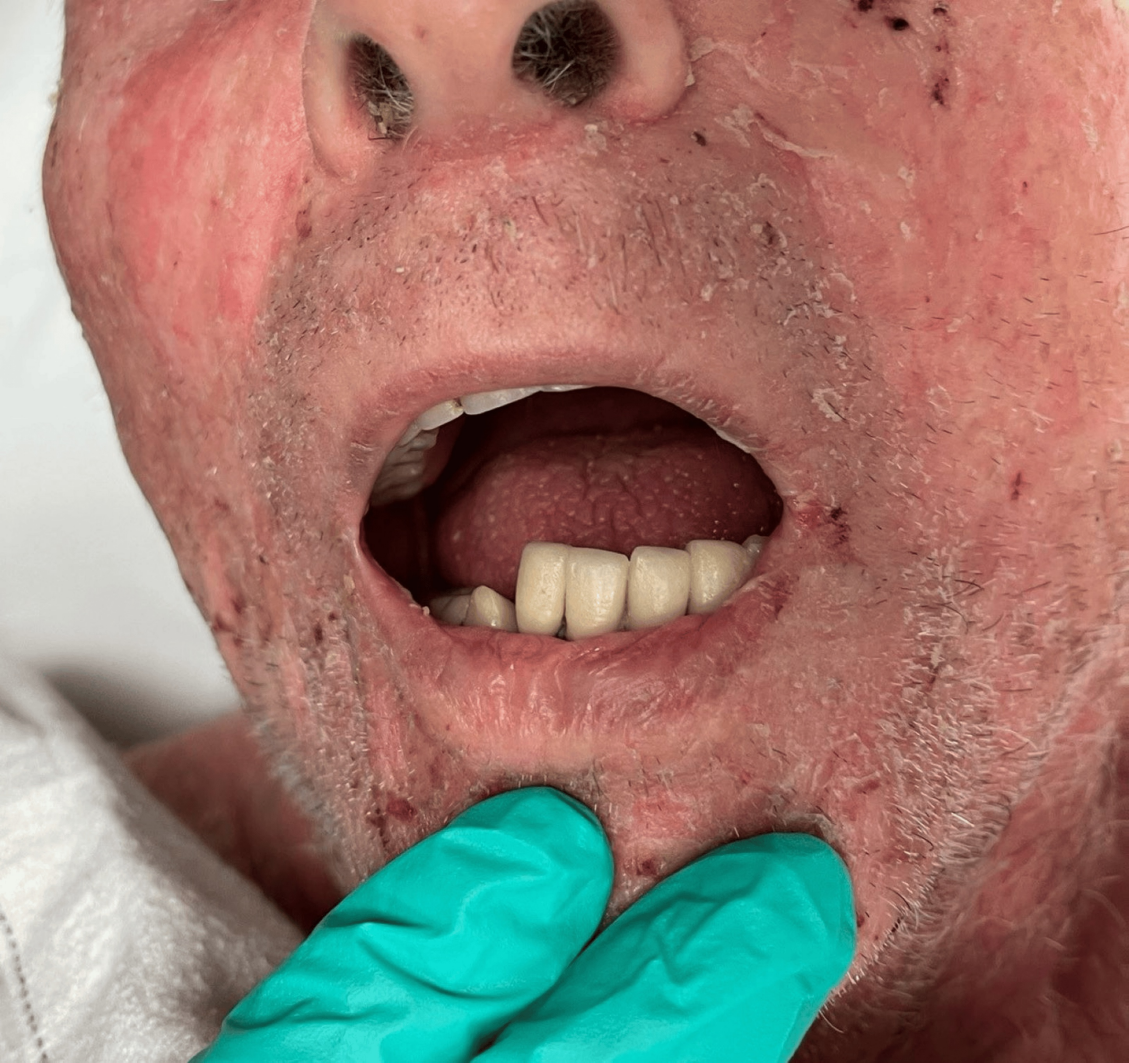
Paraneoplastic pemphigus involving the face but sparing the oral mucosa, pre-treatment.

Lost to follow-up for one year, he presented again with an intensely painful and pruritic rash covering 90% body surface area (BSA) (Figure 2). The exam showed diffuse scaling, erythema, crust, bullae, and erosions on the face, scalp, neck, trunk, and extremities, still without mucosal involvement (Figure 3). At this point, PNP, AGEP, and PF remained high on the differential, as did other causes of erythroderma including psoriasis, systemic response to scabies, seborrheic dermatitis, and cutaneous T-cell lymphoma. Repeat biopsies with DIF revealed 3+ intercellular and basement membrane deposition of C3 and IgG. Indirect immunofluorescence (IIF) was negative. Anti-plakin antibodies were unobtainable at the institution. Inpatient topical steroids provided temporary improvement before discharge.

At one month follow-up, he reported minimal improvement, with erythema involving 80% BSA and eroded heme-crusted lesions involving 10% BSA. The patient's primary concern was pruritus recalcitrant to topical corticosteroids and oral antihistamines. Given the patient's medical history of T2DM, chronic non-healing ulcers, and untreated malignancy, he was a poor candidate for first-line immunosuppressants. Dupilumab (loading dose of 600 mg and maintenance dose of 300 mg every other week) was elected to control the patient's pruritus.

**Figure 4:**
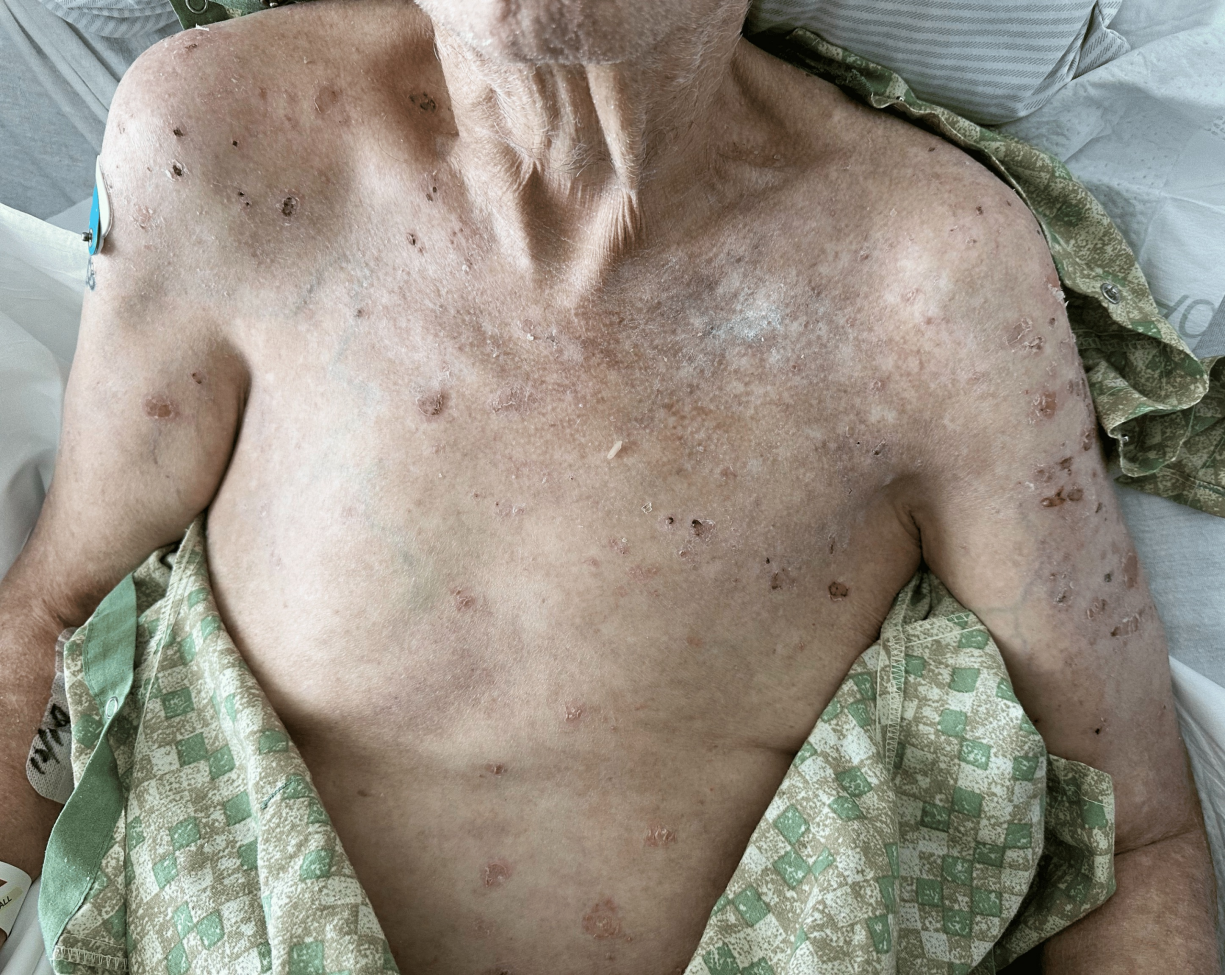
Partial improvement of paraneoplastic pemphigus involving the chest and shoulders, two months post-treatment.

**Figure 5:**
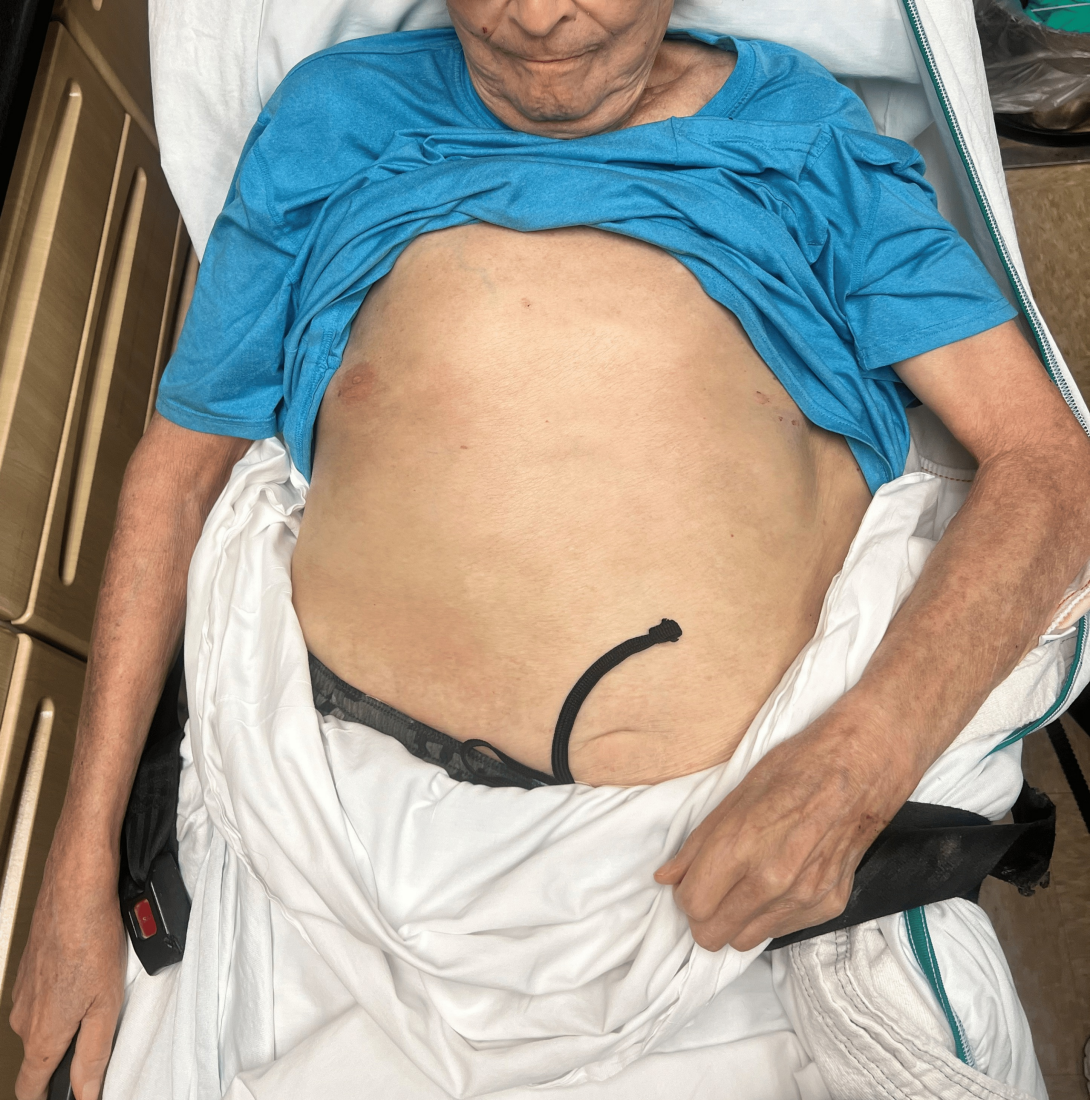
Complete resolution of paraneoplastic pemphigus involving the chest, abdomen, arms, and face, four months post-treatment.

Two months later, there was significant patient-reported improvement in pruritus and clinical appearance and number of skin erosions (Figure 4). At four months follow-up, there were complete resolution of pruritus and 100% clearance of skin lesions (Figure 5). Unfortunately, the patient expired one month after his last follow-up due to an unspecified cause of death, possibly relating to multiple medical comorbidities and known malignancy.

## Discussion

This case highlights an atypical presentation of PNP without mucosal involvement, which is often the most characteristic feature and first presenting sign [6]. Few cases of atypical PNP have been published, and its significance is unknown. One case demonstrated a significant improvement in skin lesions after treatment with clobetasol propionate 0.05% cream and anastrozole for underlying invasive breast carcinoma [3]. Considering these significant responses to treatment, it may be possible that atypical PNP confers a better prognosis. Desmoglein-3 antibodies are thought to be important for mucosal involvement, and our patient notably lacked these. However, mucosal involvement can be seen even in the absence of these antibodies [3]. More studies describing atypical PNP are needed to ascertain its clinical and prognostic significance.

The lack of mucosal lesions and initial equivocal DIF lead to a differential diagnosis initially considering AGEP or PF. AGEP is a superficial pustular eruption and delayed hypersensitivity reaction most commonly to antibiotics and antifungals [7]. PF does not involve mucosa and only has desmoglein-1 autoantibodies, while pemphigus vulgaris (PV) is mucocutaneous and has both desmoglein-1 and desmoglein-3 autoantibodies [8]. Other causes of erythroderma, including psoriasis, systemic response to scabies, seborrheic dermatitis, and cutaneous T-cell lymphoma, were subsequently ruled out by biopsy results. This case meets two major and two minor diagnostic criteria proposed by Svoboda et al. necessary for PNP diagnosis: positive serology for BPAG1/BP230 autoantibodies (a member of the plakin family) [2], underlying malignancy, DIF positive for intercellular and basement membrane staining, and acantholysis with lichenoid interface changes (Table 1) [4]. IIF can be negative in up to a quarter of cases of PNP [5].

**Table 1:** Proposed diagnostic criteria for paraneoplastic pemphigus, requiring three major or two major and both minor criteria, which were met in this case. DIF: direct immunofluorescence; ELISA: enzyme-linked immunosorbent assay; IP: immunoprecipitation; IB: immunoblot; IIF: indirect immunofluorescence Reference: [4]

Major criteria	Met	Not met
Mucous membrane lesions±cutaneous involvement		×
Concomitant internal neoplasm	×	
Evidence of anti-plakin autoantibodies, including but not limited to IP, IB, ELISA, and IIF on the transitional epithelium	×	
Minor criteria		
Acantholysis and/or lichenoid interface observed on histopathology	×	
DIF displaying intercellular and/or basement membrane staining	×	

Dupilumab is not currently approved by the Food and Drug Administration (FDA) for autoimmune blistering conditions. Case reports and a phase 2 double-blinded randomized clinical trial are investigating the use of dupilumab in bullous pemphigoid and PV [9]. Patients with PF and PV resistant to systemic and topical corticosteroid therapy achieved partial to complete responses to concurrent dupilumab and immunosuppressants [9,10]. To our knowledge, no cases have described the use of dupilumab for PNP, as we have presented with this case. A proposed mechanism may involve suppressing the T-helper-2 cell pathway involved in autoantibody production in these diseases [9].

## Conclusions

PNP is a rare autoimmune blistering disease associated with malignancy, while atypical PNP without mucosal involvement is an even more rare presentation. This report adds to the scarce literature on this rare variant. This case also draws attention to the proposed diagnostic criteria for PNP, which allows non-mucosal variants to fall under the entity of PNP. The decision to use dupilumab in this case took into consideration the complex patient factors, such as active malignancy, other comorbidities, and symptomatic control, that affect the management of PNP. However, mortality remains high in PNP patients despite treatment. Dupilumab may provide symptomatic control and improve the quality of life in these patients. Additional studies are needed to investigate dupilumab as a non-immunosuppressive adjunctive or monotherapy in patients with PNP and other autoimmune blistering diseases.
